# What changed between 2008–2020 about Employees' perception of hygiene in the catering industry in Ankara (Turkey)?

**DOI:** 10.3934/publichealth.2021021

**Published:** 2021-03-18

**Authors:** Aslı Uçar, Taha Gökmen Ülger, Funda Pınar Çakıroğlu

**Affiliations:** 1 Ankara University Faculty of Health Sciences, Department of Nutrition and Dietetics, Ankara, Turkey; 2 Bolu Abant Izzet Baysal University Faculty of Health Sciences, Department of Nutrition and Dietetics, Bolu, Turkey

**Keywords:** food safety, perception of hygiene, foodborne illness, food safety regulations, employees in the catering industry

## Abstract

Food safety is a public health concern because foodborne diseases have been increasing in recent years due to several factors such as urbanization, globalization and changes in consumer habits. Many countries in the world, including Turkey have upgraded their laws about food and personnel hygiene in the catering industry and undertaken changes to the organizational structure of their regulatory institutions to protect consumers' health. In this study, it was aimed to evaluate the perceptions of food processors on food safety and to determine whether there has been a change in this matter over the last 12 years. The data has been collected by conducting face to face interviews and having 500 employees from the sector fill in a questionnaire constructed for this purpose. The responses to the questionnaire have been measured by assigning ‘hygiene perception points’ to each respondent according to their replies. These hygiene perception points have been analysed in terms of gender, age, educational level and work experience of the employees involved. The results have revealed that employees between the ages of 26–34, women, university graduates have a higher level of perception of hygiene than other age groups, men, those with lower education levels, respectively. Hygiene perception points were found to be higher compared to the results obtained 12 years ago. The positive changes observed in the hygiene perception points are thought to result from the differences in the legislation of the years in which both studies were conducted. It is thought that the obligatory of providing hygiene and food safety training to individuals working in the catering sector with law changes leads to positive changes in the employees. Legally compulsory training activities can overcome many sanitation and safety problems that result from misinformed or uninformed employees.

## 1. Introduction

Urbanization and changes in consumer habits, including travel, have increased the number of people buying and eating food prepared in public places. Globalization has triggered growing consumer demand for a wider variety of foods, resulting in an increasingly complex and longer global food chain. As the world's population grows, the intensification and industrialization of agriculture and animal production to meet increasing demand for food creates both opportunities and challenges for food safety [1]. Food safety is considered as a global health target because foodborne diseases are major health problem in the world today [2].

Foodborne diseases can be prevented, but they are a threat for global health due to the serious illnesses and deaths they cause. There are an estimated 600 million cases of foodborne disease leading to 420,000 deaths worldwide annually [1]. Of particular concern are the deaths of children under 5 years of age with 125,000 deaths each year, 30% of the total [1]. Although children under the age of 5 are identified as a risk group for foodborne diseases, pregnant women, the elderly, individuals with the underlying disease and those who are immune-comprised such as patients undergoing chemotherapy and organ transplants are also particularly vulnerable to these diseases. Foodborne diseases risk is also higher for travelers, refugees and immigrants, because they may be exposed to unfamiliar foodborne hazards in new environments. Although many factors such as bacteria, viruses, parasites or chemicals cause foodborne illnesses, it has been stated that diarrheal disease agents are the most common cause of foodborne diseases in many countries [3]–[5]. While there is no comprehensive data on pathogens which cause food poisoning in Turkey, it has been stated that food poisoning is in the second place after drug poisoning among the cases of applying to the emergency service due to poisoning [6],[7]. On the other hand, not only developing countries, but also developed countries are at risk of foodborne diseases. According to the CDC reports, each year roughly 1 in 6 Americans (or 48 million people) gets sick, 128,000 are hospitalized, and 3,000 die of foodborne diseases [8]. In the European Region, it was estimated that more than 23 million people fall ill from eating contaminated food every year, resulting in 4654 deaths [3].

The majority of the of foodborne illnesses are derived from food services in the food production premises [9]. The most common causes of food borne poisoning cases are reported as inadequate cooling, one or more hours between preparation and consumption, incorrect heat treatment, inadequate cooking, inadequate heating, using contaminated material, cross contamination, inadequate cleaning of the equipment, using improper food materials and leftover food. In addition, as reported in some studies [10],[11] infected personnel, improper hand washing practices, insufficient cleaning of processing equipment and cross-contamination of ready-to-eat food are the most frequent errors made by professional food handlers resulting in subsequent outbreaks. It was also indicated that food handlers play a major role in contaminating food [12]. These challenges put greater responsibility on food producers and handlers to ensure food safety. Food processors, the last link in the safe reach of food to consumers, are recognised as the heart of food safety systems [13].

Inadequate knowledge, uncaring attitude, and improper practice of food handlers are key factors that have led to foodborne disease outbreaks in foodservice facilities including catering [14]. In various studies, consumers and food industry workers have been revealed to have a lack of information and negligence in terms of food preparation safety [15],[16]. Levels of food safety knowledge and knowledge gaps among professional food handlers [17], consumers [18],[19], and young consumers [20],[21] have been studied in scientific literature. Although such studies are limited in Turkey, that of Çakıroğlu & Uçar in 2008 [22] concluded that the knowledge of the employees' working in the catering industry in Ankara was inadequate. We wished to determine whether these findings were still relevant more than one decade later by studying the perceptions of employees working in the catering industry in Ankara on food safety issues.

## 2. Materials and methods

The sample of this study consists of a total of 500 volunteer employees working at nine mass catering firms registered to the Chamber of Commerce (ATO) in Ankara. We selected the same companies participating in the previous survey [22], but since employees are transient in the food industry, only some of these were participants in former study. Thus, the differences in the perception of the personnel working in the same workplaces and the situation in this sector in general have been tried to reflect.

Data were collected through face to face interviews and a questionnaire. The first 8 items of the questionnaire have been designed to obtain information about the demographic characteristics and health status of the participants. In order to determine their perception of hygiene, the 36-statement Likert type scale, whose validity and reliability were checked (alpha = 0.8290) by Buyruk & Şahin (2002) [23], has been used. In the scale, 14 statements are about “food hygiene”, 12 statements are about “personel hygiene” and 10 statements are about “kitchen and equipment hygiene”. The scale includes a set of negative sentences (statements 3, 4, 15, 17, 23, 27, 28, 32, 34 and 36) in addition to the positive ones. Responses to the positive sentences have been graded as follows: ‘I certainly agree’, 5 points; “I agree”, 4 points; “undecided”, 3 points; “I don't agree”, 2 points and “I certainly don't agree”, 1 point. In the negative sentences, the grades have been assigned in reverse order. When all the statements are replied correctly, the grade that should be obtained from the “kitchen and equipment hygiene” part is 50 points; from the “personel hygiene” part, 60 points; and from the “food hygiene” part, 70 points, amounting to a total of 180 points.

The findings have been analysed with respect to gender, educational level and work experience variables in the Statistical Package for Social Sciences (SPSS) programme. In evaluating the hygiene perception grades, “Independent-samples T test” for the gender varible, “One-way anova” analysis and “scheffe test” for the other variables have been applied. Frequencies, averages and standart deviations have been calculated.

## 3. Results

### 3.1. Sampling characteristics

Of the 500 volunteer personnel, 70.2% were male, and 29.8% were female; 36.0% were between 26–34 years of age; 49.2% were high school graduates; 56.0% were working in kitchen while the others were in service department (56.4% of them were servers, 32.2% were cooks, and 5.6% of them were dishwashers). The mean working duration of participants were 8 ± 7.8 years; in fact, 56.0% of the personnel had a service period of less than 6 years. On the other hand, most of the participants stated that they participated in at least one training activity on mass catering (73.2%), kitchen and equipment hygiene (66.4%), personal hygiene (67.6%) and food hygiene (67.8%) subjects.

### 3.2. Hygiene perception of catering staff based on gender

**Figure 1.**
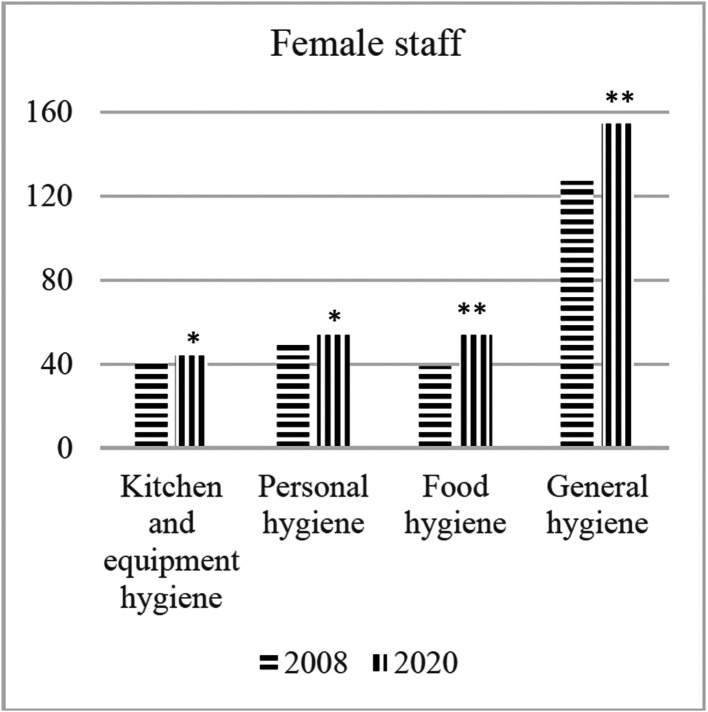
The changes in hygiene perception in female catering staff. Note: *p < 0.05. **p < 0.01.

**Figure 2.**
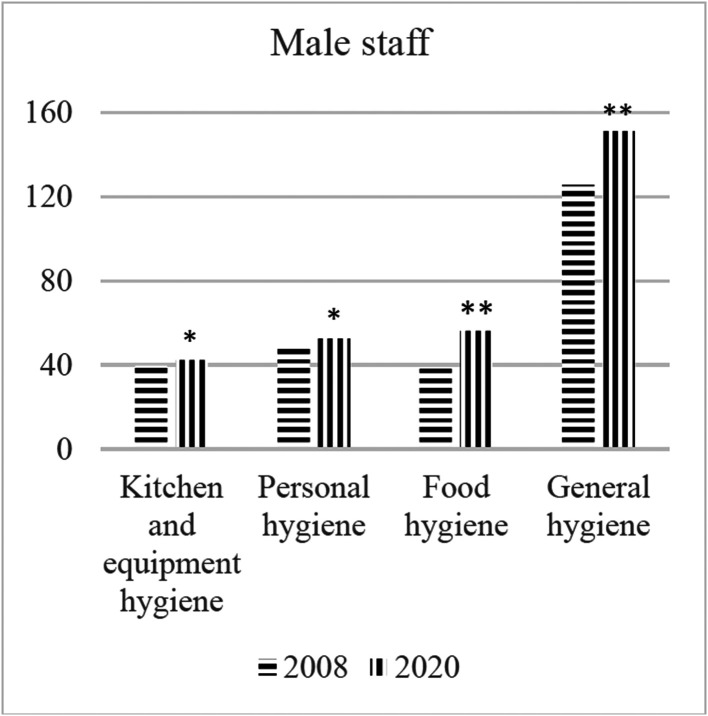
The changes in hygiene perception in male catering staff. Note: *p < 0.05. **p < 0.01.

Table 1 shows the average hygiene perception points of employees in catering firms. According to these results, women employees have received higher grades than men. The results of the statistical analysis shows that this difference is significant in “Kitchen and equipment hygiene”, “Personal hygiene” and “General hygiene” sections. In addition, hygiene perception points in all sections were found to be significantly higher compared to the results obtained 12 years ago in both genders (Figure 1 and Figure 2).

**Table 1.** The results of t test towards hygiene perception of catering staff based on gender.

	Male	Female	*t*	Sig
Kitchen and equipment hygiene	42.3 ± 6.0	43.9 ± 5.2	3.026	0.003**
Personal hygiene	52.6 ± 6.1	53.7 ± 5.0	1.966	0.049*
Food hygiene	56.1 ± 6.9	56.7 ± 5.5	1.088	0.277
General hygiene	151.1 ± 16.8	154.4 ± 13.1	2.355	0.019*

Note: *p < 0.05. **p < 0.01.

### 3.3. Hygiene perception of catering staff based on age groups

As seen in Table 2, the scores of the personnel between the ages of 26–34 are highest. The lowest score in the kitchen and personal hygiene part received by the age group 45 and above, while the lowest score in the food hygiene part received by the group under the age of 25.

**Table 2.** The results of variance analysis towards hygiene perception of catering staff based on age groups.

	<25	26–34	35–44	45+	F	Sig
Kitchen and equipment hygiene	42.7 ± 6.2	43.4 ± 5.6	42.7 ± 5.5	41.0 ± 5.9	2.376	0.069
Personal hygiene	52.6 ± 6.1	53.5 ± 5.4	52.8 ± 5.8	51.9 ± 6.8	1.255	0.289
Food hygiene	55.3 ± 6.3	57.3 ± 5.8	56.2 ± 6.9	55.5 ± 7.9	2.564	0.054
General hygiene	150.7 ± 16.1	154.3 ± 14.5	151.8 ± 15.9	148.6 ± 18.6	2.379	0.069

### 3.4. Hygiene perception of catering staff based on educational status

Considering the education level, it was seen that university graduates have the highest score in each category and primary school graduates have the lowest score (p < 0.01). In addition the scores received increased as the level of education increased (Table 3).

**Table 3.** The results of variance analysis towards hygiene perception of catering staff based on educational status.

	Primary school	Secondary school	High school	University	F	Sig	Difference
Kitchen and equipment hygiene	41.3 ± 6.0	41.1 ± 5.8	43.4 ± 5.6	46.2 ± 4.0	12.421	0.000**	1–3, 1–4, 2–3, 2–4, 3–4
Personal hygiene	52.8 ± 5.5	51.5 ± 6.3	53.0 ± 5.9	55.9 ± 3.4	6.415	0.000	1–4, 2–3, 2–4, 3–4
Food hygiene	54.8 ± 7.6	54.9 ± 7.0	57.0 ± 5.8	58.5 ± 5.2	6.484	0.000*	1–3, 1–4, 2–3, 2–4
General hygiene	149.0 ± 16.9	147.6 ± 16.7	153.6 ± 15.0	160.8 ± 10.5	10.143	0.000*	1–3, 1–4, 2–3, 2–4, 3–4

Note: *p < 0.05. **p < 0.01.

### 3.5. Hygiene perception of catering staff based on work experience

While the highest scores in the general hygiene, personal hygiene and food hygiene part were obtained by individuals working between 7–10 years, the highest score in the kitchen and equipment part was obtained by those working 3–6 years (Table 4). The scores obtained in the personal hygiene and general hygiene section differ according to the working time of individuals (*p* < 0.05, *p* < 0.01, respectively).

**Table 4.** The results of variance analysis towards hygiene perception of catering staff based on work experience.

	<2	3–6	7–10	11+	F	Sig	Difference
Kitchen and equipment hygiene	42.7 ± 5.6	43.2 ± 5.9	43.1 ± 5.9	42.0 ± 5.9	1.068	0.458	
Personal hygiene	52.9 ± 5.3	53.3 ± 6.2	53.9 ± 4.8	51.7 ± 6.7	2.877	0.036**	2–4, 3–4
Food hygiene	56.2 ± 5.7	56.6 ± 6.8	56.9 ± 5.8	55.6 ± 7.5	0.868	0.362	
General hygiene	127.8 ± 1.0	122.4 ± 1.6	128.1 ± 2.0	129.8 ± 1.3	5.092	0.002**	1–2, 2–4

Note: *p < 0.05. **p < 0.01.

## 4. Discussion

According to the results, the scores of both genders in all sections (kitchen and equipment hygiene, personal hygiene, food hygiene and general hygiene) are higher than our previous study (Çakıroğlu & Uçar, 2008) [22]. In addition, women achieved higher scores in all sections than men, and this difference is the same as in the previous study (Çakıroğlu & Uçar, 2008) [22]. The positive changes observed in the hygiene scores are thought to result from the differences in the legislation of the years in which both studies were conducted. According to the legislation valid in 2008 (Law no: 1593/126-127); feces, throat check and lung scintigraphy test was an obligatory for the staff who work in the food endustry and this law allowed employees to work in this sector only upon approval from a hospital (Bulduk, 2003) [24]. In the following years, this regulation was changed (Food Hygiene Regulation No. 28145 dated 17.12.2011) so that feces, throat control and lung scintigraphy test were no longer obligatory. With the new regulation, food operators were held responsible for “Ensuring that the food contact personnel are healthy and receive training on health risks”. The same regulation also introduced some rules for personnel hygiene. According to these rules, it is a legal obligation for the employees to wear appropriate protective clothing, pay attention to personal cleanliness, not enter the areas where food is processed in case of illness and report the disease to the employer. In addition, food operators are obliged to ensure that “staff working in the catering industry are checked and informed and/or trained on food hygiene issues required by their work” with the same legislation. After the relevant legislation came into force, employees in this sector were frequently trained on hygiene and food safety. On the other hand efforts to inform the employees in catering industry about their work and risks gained weight. As a result, it is thought that the obligatory of providing hygiene and food safety training to individuals working in the catering sector with law changes leads to positive changes in the employees.

Training is defined as “a planned process to modify attitude or skill behaviour through learning experience to achieve effective performance in an activity or range of activities” [25]. The quality of the training is also of great importance for permanent behavior changes in the staff. It is emphasized that several training activities on hygiene and food safety can be effective in increasing the level of knowledge of the personnel, but they do not have a significant effect in creating permanent behavior change [26]. In Cunha et al.'s study (2014) [27] it was stated that majority of participants (68.3%) had participated in at least one food safety training session (In Brazil, where this study is carried out, it is imperative as in Turkey that the personnel who come into contact with food be healthy and receive training on health risks). The average percentage of correct answers on the knowledge questionnaire was 64%. Food handlers who had undergone training presented higher knowledge scores but did not differ from those who had not regarding attitudes, self-reported practices and observed practices [27]. Educational efforts resulting in behaviour change are therefore an important strategy contributing to the reduction of foodborne illnesses in the food business sector (Jevšnik et al., 2008a) [16]. Food safety laws should also enable the establishment of policies to monitor and ensure the adequacy of food services.

Several factors before and after training may affect the quality of training [28]. The importance given to training by the food business operator, the use of correct and suitable educational tools and the support of colleagues can increase the effectiveness of the training, thereby ensuring that the positive behavior changes to be observed at the end of the training last longer [28]. Therefore, determining all factors that may affect the quality of training and increasing the quality of training can be effective in reducing the risk of foodborne disease. Education level is one of the main factors that determine the effectiveness of training activities. In this study, most of the food handlers stated that they participated in at least one training activity on mass catering (73.2%), kitchen and equipment hygiene (66.4%), personal hygiene (67.6%) and food hygiene (67.8%) subjects but it is clear that these training activities should be more effective. Increasing the education level of food handlers may increase the efficiency of food hygiene and food safety trainings. According to the results obtained from this study, it was seen that the scores received increased as the level of education increased (Table 3). However, in Turkey, mostly individuals with high school or less education work in this sector. The obtained results reveal the necessity of the employees of the mass nutrition system and the catering industry to be composed of educated people. The necessity to increase the level of education in this sector has been stated in many studies [29]–[31]. Another problem to consider is the high circulation rate in individuals working in this sector. In addition, it is thought that the rate of participation in the trainings may not have reached 100% since the trainings are generally given during the working hours of the employees. These problems may have reduced the effectiveness of training activities. All these factors need to be taken into account to increase the effectiveness of training activities.

Enforceable and relevant policies are required to create an enabling environment in which to develop and enforce food safety measures [32],[33]. In light of the increasing burden of foodborne diseases, international standards are becoming stricter. Many countries in the world have upgraded their laws about food and personnel hygiene in the catering industry and undertaken changes to the organizational structure of their regulatory institutions to protect consumers' health [34]. Despite all these positive developments it is not clear whether the recent changes will serve towards science-based and effective preventive functions and the adoption of the risk management approach [35]. But some evidences showed that food safety and food hygiene training in the commercial sector of the food industry can result in improved food safety [34],[36]–[39]. It is also stated that mandatory training programmes are more successful than voluntary programs [34],[40]. For this reason, it is thought that the training to be given to individuals working in this sector may be effective in preventing foodborne diseases and it may be beneficial to have this legally mandatory.

Finally, as food handlers are the center of food safety systems, their education and training are vital for food safety management [13]. Because infected personnel, improper hand washing practices, insufficient cleaning of processing equipment and cross-contamination of ready-to-eat food are the most frequent errors made by professional food handlers resulting in subsequent outbreaks [10],[11]. Educational efforts resulting in behaviour change are therefore an important strategy contributing to the reduction of foodborne illnesses in the food business sector [16]. Food safety laws should not only require certification but also enable the establishment of policies to monitor and ensure the adequacy of food services. Increasing the supply of safe and wholesome food with training activities reduces the impact of foodborne diseases that cause both human suffering and significant economic losses.

## 5. Conclusions

Many countries in the world have upgraded their laws about food and personnel hygiene in the catering industry and undertaken changes to the organizational structure of their regulatory institutions to protect consumers' health. It is clear that there is an improvement in hygiene perception scores compared to the results obtained 12 years ago. Effective and relevant food safety/hygenie training activities will have a greater effect on knowledge, and actual behaviour of the food handlers. Determining all factors that may affect the quality of training activities is an important topic and should be investigated with future studies to increase the effectiveness of training activities.
