# Supplementary material for: What changed between 2008–2020 about Employees' perception of hygiene in the catering industry in Ankara (Turkey)?
Source: AIMS Public Health. 2021 Mar 18;8(2):275–84. doi: 10.3934/publichealth.2021021 (PMC8116189; doi:10.3934/publichealth.2021021)
Supplement: Supplementary file 1 [file publichealth-08-02-021-s001.pdf]

Public Health

AIMS Public Health, 8(2): 275–284.
DOI: 10.3934/publichealth.2021021
Received: 16 January 2021
Accepted: 15 March 2021
Published: 18 March 2021

http://www.aimspress.com/journal/aimsph

Research article

What changed between 2008–2020 about Employees' perception of hygiene in the catering industry in Ankara (Turkey)?

Aslı Uçar[1], Taha Gökmen Ülger[2,*] and Funda Pınar Çakıroğlu[1]

[1] Ankara University Faculty of Health Sciences, Department of Nutrition and Dietetics, Ankara, Turkey
[2] Bolu Abant Izzet Baysal University Faculty of Health Sciences, Department of Nutrition and Dietetics, Bolu, Turkey

* **Correspondence:** Email: tahagokmenulger@ibu.edu.tr; Tel: +905321797875; Fax: +903742534557.

Appendix. The scale used in the research

1. Food hygiene is purification from the elements that cause disease
2. Each staff is a carrier of bacteria and he or she can spread it
3. There is no objection to the fact that garbage and food can be kept together in the kitchen (–)
4. Staff who are responsible for food service can touch food with bare hands (–)
5. Uncooked and cooked foods should be prepared on separate counters
6. Bacteria spread mostly through respiration
7. The outbreak of food poisoning damage the image of a catering firm
8. The part of the body on which microorganisms can be mostly found is hand
9. Bacteria can be spread through open wounds and digestive system
10. Staff health check-up should be conducted in every six months
11. Hot meals should be kept at 60 °C or higher until they are served
12. There is objection to the fact that employees can enter the kitchen in their shoes or clothes they wear outside (–)
13. Employees should wear clean and ironed caps or bonnets
14. The inside of cold stores and refrigerators should be clean and well-cared
15. There is no objection to the fact that sunlight can enter into the store or pantry (–)
16. Grounds of the fields of food production and service should be well-cared and kept dried

17. There is no objection to the fact that products with milk and egg and meat products can be kept at high temperature (–)
18. When purchasing meat, one should pay attention to the fact that it has labels
19. One should not put his or her hands into water glasses
20. There should be enough showers and bath for the staff
21. In case of cold, flu, diarrhea etc., staff should not work or they should work in background service
22. Clean and healthy water analysed in a laboratory should be used in the kitchen
23. There is no objection to the fact that one can hold forks and knives from their top parts (–)
24. Washing hands hygienically means washing them with hot water and soap by rubbing them from wrists and by brushing nails
25. Staff should pay attention to hygiene while entering and exiting the working area
26. The surfaces where food is prepared should be cleaned after putting away the foods
27. There is no objection to the fact that staff can smoke (–)
28. The used cleaning clothes do not always need to be washed and dried (–)
29. Hot food should be stored and cooled in small and shallow containers
30. In the course of washing dishes, one should be fastidious while washing, rinsing and sterilization of the dishes
31. Wastes should be eliminated from the kitchen by grinding or putting in the waste baskets
32. There is no objection to the fact that cooked food can be kept for two hours before serving them (–)
33. Wounds on the hands of the staff should be covered by water proof bandages
34. Food that has been left outside the fridge longer than 2 hours after dissolving can be refrozen (–)
35. Food should be tasted by a spoon different from the one used for stirring it
36. Frozen food should be thawed in a warm environment (in the kitchen, on the radiator etc.) (–)

Note: Variables numbered 1, 3, 4, 5, 7, 11, 17, 18, 22, 29, 32, 34, 35, 36 are about "Food hygiene". Variables numbered 2, 6, 8, 9, 10, 13, 20, 21, 24, 25, 27, 33 are about "Personel hygiene". Variables numbered 12, 14, 15, 16, 19, 23, 26, 28, 30, 31 are about "Kitchen and equipment hygiene".

AIMS Press

© 2021 the Author(s), licensee AIMS Press. This is an open access article distributed under the terms of the Creative Commons Attribution License (http://creativecommons.org/licenses/by/4.0)
